# Factors associated with emergency services use in Taiwanese advanced cancer patients receiving palliative home care services during out-of-hours periods: a retrospective medical record study

**DOI:** 10.1186/s12904-018-0302-8

**Published:** 2018-03-12

**Authors:** Yee-Hsin Kao, Yao-Ting Liu, Malcolm Koo, Jui-Kun Chiang

**Affiliations:** 1 Department of Family Medicine, Tainan Municipal Hospital, Tainan, Taiwan; 2 Department of Medical Research, Dalin Tzu Chi Hospital, Buddhist Tzu Chi Medical Foundation, Dalin, Chiayi, Taiwan; 3 Dalla Lana School of Public Health, University of Toronto, Toronto, Ontario Canada; 4 Division of Family Medicine, Dalin Tzu Chi Hospital, Buddhist Tzu Chi Medical Foundation, Dalin, Chiayi, Taiwan

**Keywords:** Palliative care, Terminal illness, Cancer, Emergency department

## Abstract

**Background:**

For patients receiving palliative home care, the need to visit the emergency department is considered to be an indicator of poor quality care. The situation can be particularly distressing when it occurs outside of normal hours of palliative home care service. The aim of this study was to investigate the factors for emergency department use during out-of-hours periods of palliative home care service among advanced cancer patients in Taiwan.

**Methods:**

This case-control study was based on a retrospective medical chart review (January 2010 to December 2012) of advanced cancer patients who were receiving palliative home care in a community hospital in south Taiwan. The use of emergency medical services by these patients was dichotomized into either normal hours (8 a.m. to midnight, Monday to Friday, excluding public holidays) of palliative home care or outside normal hours. Logistic regression analyses were performed to evaluate factors associated with emergency services use during out-of-hours period of palliative home care.

Results: Of the 94 patients receiving palliative home care, 65 had used emergency services at least once during the 3-year study period. Of these 65 patients, 40% used emergency services during out-of-hours of palliative home care. Patients with distressing conditions (defined as the occurrence of any two conditions of dyspnea, change of consciousness, or gastrointestinal bleeding) were significantly more likely to use emergency services during out-of-hours of palliative home care.

**Conclusions:**

Patients at risk of developing dyspnea, change of consciousness, or gastrointestinal bleeding should be provided with relevant information regarding these symptoms and signs.

## Background

Despite advances in early diagnosis and treatment of cancer, it remains a major cause of mortality worldwide [1]. Therefore, end of life care is a crucial aspect of cancer management. It has been shown that many patients with cancer, when asked, express a wish to die at home [2], and Taiwanese patients are of no exception. A survey in Taiwan indicated that 61% of patients with advanced cancer preferred to die in their own homes [3]. In traditional Chinese culture, dying at home means that the spirit of the dead has a place to rest in peace. Conversely, out-of-home death is thought to bring bad fortune for the deceased in the afterlife [4, 5]. A population-based study on the specific locations of cancer deaths in Taiwan during 1997 to 2003 showed that approximately 60% of all cancer deaths occurred at home [6]. Patients, at their last moment of life, are often provided with artificial respiratory support to allow them to die at home instead of hospitals. In addition, people dying in institutions were found to have more unmet needs for symptom amelioration, physician communication, emotional support, and being treated with respect at the end of life, compared with those receiving palliative home care [7]. Therefore, dying at one’s own home is considered as a good death.

The use of palliative home care can facilitate effective care of dying patients at home. Improved quality of life in patients receiving palliative home care has been reported [8, 9]. In addition, a population-based study, using the Taiwan’s National Health Insurance Research Database from 2009 to 2011, found that patients dying from cancer with palliative home care were associated with US$2452 less in expenditure per patient compared with those without the service [10].

Visits to emergency services made near the end of life are considered as one of the indicators of poor-quality cancer care [11]. Therefore, it could be useful in identifying factors associated with emergency services use in patients with advanced cancer [12]. In Taiwan, currently there are 46 hospitals with inpatient hospice ward and they can provide around-the-clock counseling phone care after patients are discharged back to home. In addition, there are 20 hospitals without inpatient hospice care beds but provide palliative home care services [13]. Advanced cancer patients in these hospitals are admitted to appropriate medical divisions according to cancer type. If these patients or their family members require palliative care advice after the patients are discharged home from hospital, a palliative home care services hotline is available. In addition, home visits by the palliative care team can be arranged if required during normal working hours. However, because of limited resources in these hospitals (including the hospital of the present study), no phone advice services or home visits are available during the out-of-hours periods. In other words, no advice can be obtained between midnight and 8 a.m. in the morning during weekdays as well as whole day during weekends and public holidays. Under such conditions, some patients may have to resort to the use of emergency service, which is a highly distressing experience for both the patients and their family members. Nevertheless, it is not clear what patients’ characteristics are more likely to use emergency services outside of normal hours. Therefore, the aim of this study was to investigate factors associated with emergency services use during the out-of-hours periods of home palliative care in advanced cancer patients.

## Methods

### Study design and subjects

This case-control study was based on a retrospective medical chart review of advanced cancer patients who were enrolled in the palliative home care program in a community hospital in southern Taiwan. The hospice shared-care model was adopted in Taiwan since 2005. Under the model, patients can receive palliative service by a specialized team without the need to leave their original medical care team and environment in hospital acute ward. In addition, if patients’ condition is stable and they wants to go home, they will be discharged and continue to receive palliative care at their own home.

**Fig. 1:**
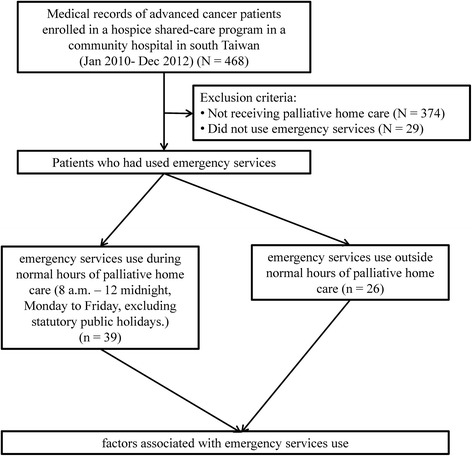
Study flowchart

The study flowchart is shown in Fig. 1. A total of 468 patients were identified and 374 of them did not receive hospice shared-care. Of the remaining 94 patients, 29 did not use emergency services during the study period. Therefore, 65 patients were included in this study. The use of emergency medical services by these patients at the study hospital from January 2010 to December 2012 was ascertained from medical records. The study protocol, including medical record review, was approved by the institutional review board of the Tainan Municipal Hospital, Taiwan (SCMH_IRB No: 1021005).

### Measurements

The following information was ascertained from the medical records: sex, age, marital status, educational level, number of people living together, location of residence, type of cancer, type of caretaker, length of palliative home care service, number of times of home visits, Eastern Cooperative Oncology Group (ECOG) performance status, and home death. In addition, variables related to emergency service use were also collected and they included: mean number of emergency services use, mean number of hospitalizations, clinical symptoms or signs when admitted to emergency services, and mean daily oral morphine equivalent dose prior to emergency admission.

The location of the residence was classified into urban versus suburban and rural levels of urbanization [14]. ECOG performance status scores were categorized as grade 3 when patients were capable of only limited self care and confined to bed or chair more than 50% of waking hours; and as grade 4 when patients were completely disabled, could not carry on any self care, and totally confined to bed or chair.

Seven common clinical symptoms or signs of advanced cancer patients including pain, infection or fever, nausea or vomiting, constipation, dyspnea, change of consciousness, and gastrointestinal bleeding were recorded at the time of emergency admission. A composite variable of distressing conditions for patients and caretakers was created by simultaneously considered dyspnea, change of consciousness, and gastrointestinal bleeding. When two of these three symptoms or signs were present, distressing conditions were defined as occurring. We selected these symptoms and signs because these conditions are generally difficult to handle at home.

The mean daily oral morphine equivalent dose was estimated by the amount used in the first day of admission. Home death was defined following the convention of previous studies [15]. When the date of discharge was the same as that of death, death was considered to occur in the hospital. Otherwise, it was considered as home death.

The main binary outcome variable of this study was the use of emergency services (at the time of emergency admission) during out-of-hours periods of home palliative care services versus that during normal hours of home palliative care services. Normal hours of home palliative care services were defined as 8 a.m. to midnight, Monday to Friday, excluding statutory public holidays.

### Statistical analysis

The statistical software R, version 3.0.1 (R Core Team, 2013) [16] was used for all statistical analyses. The level of statistical significance was set at 0.05 and all tests were two-tailed. The group differences in the basic characteristics of the patients were analyzed using Fisher’s exact test and Wilcoxon rank-sum test, as appropriate. Univariate and multiple logistic regression analyses were performed to evaluate factors associated with the use of emergency services during out-of-hours period of palliative home care.

## Results

Table 1 shows the characteristics of the patients. Of the 65 patients, 40% used emergency services during out-of-hours of palliative home care. None of the variables were significantly different between those using emergency services during normal or during out-of-hours of palliative home care. Table 2 shows the distribution of variables related to emergency services use. The mean number of hospitalization was significantly higher (*p* = 0.033) in patients who used emergency services during out-of-hours of palliative home care (range: 0–3 versus 0–1 during normal working hours).

**Table 1:** Basic characteristics of the advanced cancer patients receiving palliative home care (*N* = 65)

Variable	n (%^a^)			*p* value
	All patients	Use of emergency services^b^		
	65 (100)	During normal hours of palliative home care 39 (60.0)	During out-of-hours of palliative home care 26 (40.0)	
Sex				0.139
Male	35 (54.8)	24 (61.5)	11 (42.3)	
Female	30 (46.2)	15 (38.5)	15 (57.7)	
Mean age (SD)	72.6 (12.1)	72.1 (13.3)	73.3 (10.4)	0.888
Marital status				0.547
Being married	59 (90.8)	34 (87.2)	25 (96.2)	
Single	3 (4.6)	2 (5.1)	1 (3.8)	
Divorced	3 (4.6)	3 (7.7)	0 (0)	
Educational level				0.179
Illiterate	23 (35.4)	11 (28.2)	12 (46.2)	
Elementary school	14 (21.5)	11 (28.2)	3 (11.5)	
Junior high school or above	28 (43.1)	17 (43.6)	11 (42.3)	
Mean number of people living together (SD)	2.1 (1.4)	2.0 (1.3)	2.2 (1.5)	0.690
Urbanization level of residence				0.776
Urban	17 (26.2)	11 (28.2)	6 (23.1)	
Suburban or rural	48 (73.8)	28 (71.8)	20 (76.9)	
Type of cancer				
Liver	18 (27.7)	11 (28.2)	7 (26.9)	0.910
Lung	12 (18.5)	9 (23.1)	3 (11.5)	0.334
Colorectal	14 (21.5)	8 (20.5)	6 (23.1)	0.805
Other	21 (32.3)	11 (28.2)	10 (38.5)	0.426
Caretaker				
In-home nurse only	16 (24.6)	12 (30.8)	4 (15.4)	0.241
Spouse only	27 (41.5)	14 (35.9)	13 (50.0)	0.309
In-home nurse and spouse	10 (15.4)	4 (10.3)	6 (23.1)	0.181
Others	12 (18.5)	9 (23.1)	3 (11.5)	0.334
Mean length of palliative home care service, days (SD)	43.5 (37.4)	41.6 (35.4)	46.3 (40.7)	0.763
[median (minimum–maximum)]	[31 (3–156)]	[27 (3–139)]	[37 (3–156)]	
No. of home visit by home palliative care team (SD)	3.7 (3.9)	3.6 (3.3)	4.0 (4.7)	0.989
[median (minimum–maximum)]	[3 (1–24)]	[3 (1–15)]	[3 (1–24)]	
ECOG performance status				0.128
3	34 (52.3)	17 (43.6)	17 (66.4)	
4	31 (47.7)	22 (56.4)	9 (34.6)	
Home death				0.999
yes	13 (20.0)	8 (20.5)	5 (19.2)	
no	52 (80.0)	31 (79.5)	21 (80.8)	

*SD* standard deviation, *ECOG* Eastern Cooperative Oncology Group

^a^Percentages are column percentages except in the header row where they are row percentages

^b^Normal hours of palliative home care are defined in this study as 8 a.m. to midnight, Monday to Friday, excluding statutory public holidays. Out-of-hours of palliative home care are defined as any time outside normal hours

**Table 2:** Emergency services use in advanced cancer patients during out-of-hours periods of palliative home care (N = 65)

Variable	Use of emergency services^a^ n (%)		*p* value
	During normal hours of palliative home care 39 (60.0)	During out-of-hours of palliative home care 26 (40.0)	
Mean number of emergency services use (SD)	1.5 (1.0)	1.8 (1.6)	0.393
[median (minimum–maximum)]	[1 (1–6)]	[1 (1–9)]	
Mean number of emergency hospitalization	0.8 (0.4)	1.0 (0.4)	0.033
(SD) [median (minimum–maximum)]	[1 (0–1)]	[1 (0–3)]	
Clinical symptoms or signs at emergency admission			
Pain	33 (84.6)	14 (53.8)	0.010
Infection or fever	21 (53.8)	10 (38.5)	0.311
Nausea or vomiting	8 (20.5)	4 (15.4)	0.749
Constipation	5 (12.8)	3 (11.5)	> 0.999
Dyspnea	17 (43.6)	14 (53.8)	0.456
Change of consciousness	7 (17.9)	9 (34.6)	0.150
Gastrointestinal bleeding	5 (12.8)	7 (26.9)	0.197
Composite distressing condition^b^	4 (10.3)	8 (30.8)	0.052
Mean daily oral morphine equivalent dose prior to emergency admission (SD)	34.0 (52.0)	19.4 (28.9)	0.054
[median (minimum–maximum)]	[15 (0–300)]	[7.5 (0–120)]	

*SD* standard deviation

^a^Normal hours of palliative home care are defined in this study as 8 a.m. to midnight, Monday to Friday, excluding statutory public holidays. Out-of-hours of palliative home care are defined as any time outside normal hours

^b^The composite distressing condition variable was defined as the occurring of two of the following three symptoms and signs: dyspnea, change of consciousness, and gastrointestinal bleeding

Regarding the top three clinical symptoms or signs for seeking emergency services were pain, infection or fever, and dyspnea in both groups of the patients. However, only pain was significantly different between the two groups (*p* = 0.010). The proportion of patients consulting for pain was higher during normal working hours (84.6%) compared with that during out-of-hours of palliative home care services (53.8%). The proportion of patients presented with the composite variable of distressing condition was higher during out-of-hours (30.8%) compared with that during normal hours of palliative home care services (10.3%), with marginally statistical significance (*p* = 0.052). In addition, the mean daily oral morphine equivalent dose prior to emergency admission was higher in patients with emergency services use during normal hours of palliative home care services (*p* = 0.054).

In the univariate logistic regression of factors associated with emergency services use in advanced cancer patients during out-of-hours periods of palliative home care, only pain (*p* = 0.009) and the composite variable of dyspnea, change of consciousness, and gastrointestinal bleeding (*p* = 0.045) was significant (Table 3). Similar results were obtained in the multiple logistic regression analysis. The adjusted odds ratios of pain and the composite variable were 0.18 (*p* = 0.006) and 4.91 (*p* = 0.028), respectively (Table 4). Patients suffering from pain were less likely to use emergency services during out-of-hours of palliative home care. On the other hand, those who suffered from distressing conditions (any two conditions of dyspnea, change of consciousness, and gastrointestinal bleeding) were significantly more likely to use emergency services during out-of-hours of palliative home care.

**Table 3:** Univariate logistic regression analyses of factors associated with emergency services use in advanced cancer patients during out-of-hours periods of palliative home care (*N* = 65)

Variable	Odds ratio	95% CI	*p* value
Sex (male versus female)	0.46	0.16 to 1.25	0.130
Age (year)	1.01	0.97 to 1.05	0.693
Type of cancer			
Liver	0.94	0.30 to 2.83	0.910
Colorectal	0.43	0.09 to 1.65	0.249
Lung	1.16	0.34 to 3.85	0.805
Other	1.59	0.55 to 4.61	0.388
ECOG performance status (level 4 versus level 3)	0.41	0.14 to 1.12	0.088
Urbanization level of residence (urban versus suburban or rural)	0.76	0.23 to 2.36	0.645
Caretaker			
In-home nurse only	0.41	0.10 to 1.36	0.166
Spouse only	1.79	0.65 to 4.97	0.260
In-home nurse and spouse	2.63	0.67 to 11.34	0.170
Other	0.43	0.09 to 1.65	0.249
Clinical symptoms or signs			
Pain	0.21	0.06 to 0.66	0.009
Infection or fever	0.54	0.19 to 1.46	0.226
Nausea or vomiting	0.70	0.17 to 2.54	0.603
Constipation	0.89	0.17 to 3.98	0.878
Dyspnea	1.51	0.59 to 4.15	0.418
Change of consciousness	2.42	0.77 to 7.90	0.132
Gastrointestinal bleeding	2.51	0.70 to 9.52	0.159
Composite distressing condition^a^	3.89	1.07 to 16.24	0.045
Mean daily oral morphine equivalent dose prior to emergency admission	0.99	0.97 to 1.00	0.224

*95% CI* 95% confidence interval, *ECOG* Eastern Cooperative Oncology Group

^a^The composite distressing condition variable was defined as the occurring of two of the following three symptoms and signs: dyspnea, change of consciousness, and gastrointestinal bleeding

**Table 4:** Multiple logistic regression analysis of factors associated with emergency services use in advanced cancer patients during out-of-hours periods of palliative home care (*N* = 65)

Variable	Adjusted odds ratio	95% CI	*p* value
Pain	0.18	0.05–0.59	0.006
Composite distressing condition^a^	4.91	1.25–22.39	0.028

*95% CI* 95% confidence interval

^a^The composite distressing condition variable was defined as the occurring of two of the following three symptoms and signs: dyspnea, change of consciousness, and gastrointestinal bleeding

Nagelkerke R^2^ = 0.236

Hosmer-Lemeshow test, *p* = 0.234

## Discussion

In this medical record review study of advanced cancer patients who were receiving palliative home care, we found that pain was inversely associated with the use of emergency services during out-of-hours periods of palliative home care. In other words, patients suffering from pain were more likely to visit the emergency services during normal hours. One explanation for this observation could be that patients and their families usually are familiar with how to deal with pain, such as the use of oral morphine or transdermal fentanyl patch, and therefore able to postpone the emergency visit until the next morning. On the other hand, it is not possible for family members to deal with dyspnea, change of consciousness, or gastrointestinal bleeding at home. This is indeed the situation observed in our study. A distressing condition, defined by the occurrence of any two conditions of dyspnea, change of consciousness, or gastrointestinal bleeding, significantly increased the risk of emergency services use during the out-of-hours periods of palliative home care services. When these serious symptoms and signs occurred, regardless of the time of day or night, there are no alternatives but to go to the emergency room.

Previous research indicated that the most common complaints in patients with advanced cancer at emergency admission were pain, dyspnea, fever, nausea or vomiting, confusion, and weakness [17–19]. Our study also showed that pain, infection or fever, and dyspnea were the most frequent symptoms and signs at emergency admissions.

The results of this study should be interpreted under the context of the government-run, single-payer, universal health coverage scheme in Taiwan implemented since 1995. Patients with cancer in Taiwan are eligible to apply for a “catastrophic illness certificate” and holders of the certificate are exempt from all medical payments related to their cancer treatment. Therefore, patients’ financial issues should not be a major barrier for end-of-life care. A retrospective cohort study in Taiwan showed that the proportion of patients with cancer who had more than one emergency room visits in the last month of life increased from 15.7% in 2000 to 21.0 in 2006 [20].

This study also found that the proportion of home death was similar between both groups of emergency services users. Overall, 20.0% of the patients died at home. This figure is low compared with the 60.8% reported in a study on 269 patients receiving hospice home care provided by a hospital in central Taiwan [21]. One possible explanation for the difference lies in the definition of home death. In our study, when the date of discharge was the same as the date of death, it was considered to be a hospital death, regardless of the actual place of death [15]. If the dates of discharge and death are allowed to be the same, an additional 17 patients can be considered as having a home death. In other words, the proportion of home death will be 48% (13 + 17 patients), which is close to the 60% reported by the aforementioned study.

Several limitations in this study should be noted. First, this study employed a retrospective medical record review, which carries the limitations inherent to this type of study design. Second, pain was only recorded as a dichotomous variable. No information on pain intensity is available. Finally, the sample size is limited by the number of the patients enrolled in the home palliative care services offered by our hospital.

## Conclusion

This medical records review study found that patients with advanced cancer were significantly more likely to use emergency services during the out-of-hours periods of palliative home care if they had distressing conditions (occurrence of any two conditions of dyspnea, change of consciousness, or gastrointestinal bleeding). On the other hand, patients who had pain were more likely to use emergency services during normal hours of palliative home care service. The findings suggested that dyspnea, change of consciousness, and gastrointestinal bleeding are highly distressing conditions for patients and their families, which can lead to the use of emergency services during the out-of-hours periods of palliative home care. Palliative home care team should pay particular attention to patients that are at risk of developing these distressing conditions and provide relevant information to them and their family members regarding when emergency services should be used. Future studies may investigate the cost-effectiveness of provision of full-time counseling phone services in reducing emergency visits after midnight in advanced cancer patients.

## Abbreviations

95% CI: 95% confidence interval
ECOG: Eastern cooperative oncology group
SD: Standard deviation
